# The D519G Polymorphism of Glyceronephosphate O-Acyltransferase Is a Risk Factor for Familial Porphyria Cutanea Tarda

**DOI:** 10.1371/journal.pone.0163322

**Published:** 2016-09-23

**Authors:** Colin P. Farrell, Jessica R. Overbey, Hetanshi Naik, Danielle Nance, Gordon D. McLaren, Christine E. McLaren, Luming Zhou, Robert J. Desnick, Charles J. Parker, John D. Phillips

**Affiliations:** 1 Department of Medicine, Hematology Division, University of Utah School of Medicine, Salt Lake City, Utah, United States of America; 2 Population Health Science and Policy, Mt. Sinai School of Medicine, New York City, New York, United States of America; 3 Genetics and Genomics Sciences, Mt. Sinai School of Medicine, New York City, New York, United States of America; 4 Veterans Affairs, Long Beach Healthcare System, Long Beach, California, United States of America; 5 Medicine, Division of Hematology/Oncology, University of California Irvine, Irvine, California, United States of America; 6 Epidemiology, University of California Irvine, Irvine, California, United States of America; 7 Pathology, University of Utah School of Medicine, Salt Lake City, Utah, United States of America; CINVESTAV-IPN, MEXICO

## Abstract

Both familial and sporadic porphyria cutanea tarda (PCT) are iron dependent diseases. Symptoms of PCT resolve when iron stores are depleted by phlebotomy, and a sequence variant of *HFE* (C282Y, c.843G>A, rs1800562) that enhances iron aborption by reducing hepcidin expression is a risk factor for PCT. Recently, a polymorphic variant (D519G, c.1556A>G, **rs11558492**) of glyceronephosphate O-acyltransferase (*GNPAT*) was shown to be enriched in male patients with type I hereditary hemochromatosis (*HFE* C282Y homozygotes) who presented with a high iron phenotype, suggesting that GNPAT D519G, like HFE C282Y, is a modifier of iron homeostasis that favors iron absorption. To challenge this hypothesis, we investigated the frequency of GNPAT D519G in patients with both familial and sporadic PCT. Patients were screened for GNPAT D519G and allelic variants of HFE (both C282Y and H63D). Nucleotide sequencing of uroporphyrinogen decarboxylase (*URO-D*) identified mutant alleles. Patients with low erythrocyte URO-D activity or a damaging URO-D variant were classified as familial PCT (fPCT) and those with wild-type URO-D were classified as sporadic PCT (sPCT). GNPAT D519G was significantly enriched in the fPCT patient population (*p* = 0.0014) but not in the sPCT population (*p* = 0.4477). Both HFE C282Y and H63D (c.187C>G, rs1799945) were enriched in both PCT patient populations (*p*<0.0001) but showed no greater association with fPCT than with sPCT. *Conclusion*: GNPAT D519G is a risk factor for fPCT, but not for sPCT.

## Introduction

The cutaneous photosensitivity of PCT results from abnormally low hepatic URO-D activity.[1] Viewed stepwise, URO-D is the fifth of eight enzymes that participate in the heme biosynthetic pathway, and its function is to catalyze the conversion of uroporphyrinogen III to coproporphyrinogen III by the sequential removal from the tetrapyrrole of four carboxyl groups. The block in hepatic metabolism caused by URO-D deficiency leads to accumulation within the liver of byproducts of heme synthesis (porphyrins) that subsequently enter the plasma. Porphyrins are photosensitive molecules that upon exposure to certain wavelengths of light, present in sunlight, release energy through photon emission and formation of reactive oxygen species that can damage tissues.[2] Consequently, the clinical manifestations of PCT (skin fragility and painful blisters) affect sun-exposed areas.[1]

Among the porphyrias, PCT is unique because the majority of cases are not the result of inherited mutations of the defective enzyme. Rather PCT is primarily an acquired disease that arises as a consequence of formation of an inhibitor of URO-D (sPCT). Further, in cases in which there is a heterozygous mutation of *URO-D* (fPCT), the disease phenotype is observed only if the functional activity of the wild-type enzyme is inhibited. (In the homozygous or compound heterozygous state, mutant *URO-D* causes hepatoerythropoietic porphyria, a rare, clinically severe, congenital, cutaneous porphyria.) We have identified a porphomethene as the inhibitor of URO-D, and formation of the inhibitor is an iron dependent process.[3] Although, other unidentified physiologically relevant URO-D inhibitors may exist. Other factors including hepatitis C (HCV) infection, excess alcohol consumption, and therapeutic estrogens, in women,[4] increase the risk of developing PCT, but the importance of iron in the pathophysiology of the disease is underscored by the observation that symptoms resolve and plasma porphyrin levels return to normal when iron stores are depleted by therapeutic phlebotomy.[5, 6] Thus PCT is an iron-dependent disease and genetic variations of *HFE* (C282Y and H63D) that increase iron absorption by reducing expression of hepcidin are risk factors for developing PCT.[7–9]

A recent study by McLaren et al. identified a sequence variant, D519G (**rs11558492)**, of *GNPAT* that was associated with a high iron phenotype at presentation in men with hereditary hemochromatosis who were homozygous for *HFE* C282Y.[10] Further investigations suggested that GNPAT, like HFE, participates in the regulation of hepcidin expression.[10] These observations led us to the hypothesis that, analogous to *HFE* C282Y and H63D, *GNPAT* D519G is a risk factor for PCT. The studies reported herein support this hypothesis and substantiate the concept of *GNPAT* D519D as genetic modifier of diseases of iron metabolism.

## Materials and Methods

Patient samples were contributed by investigators participating in the Porphyria Consortium of the Rare Diseases Clinical Research Network (www.rarediseasesnetwork.org/porphyrias). All enrolled patients had clinical and biochemical evidence of PCT including typical skin lesions and elevated concentrations of urine uroporphyrin. Following informed consent, DNA samples were prepared from peripheral blood of patients according to the guidelines of a protocol approved by the Institutional Review Board (IRB) of the University of Utah School of Medicine and Mt. Sinai School of Medicine. All participants were provided with a copy of the IRB approved Informed Consent Document explaining the research study and only subjects providing written consent were studied further. All research was conducted under principles of the Declaration of Helsinki. Information on risk factors was obtained either from the results of a questionnaire completed by PCT patients enrolled in the Porphyria Consortium sponsored longitudinal study of the natural history of the porphyrias or by reviewing the medical record of patients enrolled in a University of Utah sponsored Longitudinal Study of the Porphyrias, 7201.

*HFE* sequence variants C282Y (c.845G>A) and H63D (c.187C>G) were identified using high resolution DNA melting analysis.[11, 12] PCR cycling was performed using a Realplex^2^ (Eppendorf), melting analysis was performed on a LightScanner (Idaho Technologies, Salt Lake City, UT), and melting curves were analyzed by using uAnalyze.[13] Buffer for PCR amplifications used 5X-PCR Master Mix consisting of 250 mmol/L Tris (pH 8.3), 2500 μg/mL bovine serum albumin, 15 mmol/L MgCl_2_, 250 nmol/L of each deoxynucleotide triphosphate, 2.0 units KlenTaq polymerase (AB Peptides, St. Louis, MO), 440 ngTaqStart antibody (Clontech, Mountain View, CA), 0.5x LCGreen Plus (Idaho Technologies, Salt Lake City, UT). Conditions for PCR of HFE were 94° C, 15 seconds, one cycle; denaturation at 94° C for five seconds, annealing at 60° C for five seconds, extension at 72° C for five seconds, 40 cycles. Melting conditions for identification of SNP’s was performed on completion of the PCR amplification by melting the product using the following conditions; 45° C 15 seconds followed by 45° C to 90° C at a rate of 1° C/minute. PCR primers for HFE H63D: Forward (CTTGTTTGAAGCTTTGGGCTAC,(0.1 μM final)), reverse (GAAACCCATGGAGTTCGGG (0.5 μM final)), with a melting probe of (GTTCGTGTTCTATGATGATGAGAGTCA* PO_3_^2−^ (0.4 μM final)). PCR primers for HFE C282Y forward (TGGGGAAGAGCAGAGATATAC (0.5 μM final)), reverse (TGGGTGCTCCACCTG (0.5 μM final)). The variant associated with the C282Y is detected without the need for a melting analysis probe.

*GNPAT* D519G (c.1556A>G) was identified using a validated TaqMan SNP assay (assay number C__25761550_10).[14] Nucleotide sequencing of *URO-D* included all exons, all intron-exon boundaries, and the 5’ non-coding region was performed in a CLIA certified lab at Mt. Sinai Medical Center as part of the Porpyhria Consortium’s longitudinal natural history study of the porphyrias.[15] A variant call file (VCF) was generated using the NHLBI Exome Sequencing Project exome variant server and appended to include rare clinical variants.[16] The VCF file was annotated using ANNOVAR.[17] Patients were classified as having fPCT if they were found to have low erythrocyte URO-D activity, a mutation in the *URO-D* gene predicted to be deletorious by FATHMM, or both.[18]

### Statistical Analysis

Demographic and risk factor data are displayed as mean (± standard deviation) for continuous variables and as proportions for categorical variables. Enrichment of specific genotypes was assesed by comparing the observed sample allele frequency to the frequency of the European (non-Finnish) Exome Aggregation Consortium (EXAC), a database composed of an aggregation of more than 60,000 exomes, using chi-square goodness of fit tests.[19] Allele frequency information from the EXAC database for the sub-population (non-Finnish) Europeans was used for statistical comparision as it most closly matches the demographic makeup of the study population. Associations of GNPAT, HFE C282Y and H63D genotypes with PCT type (sporadic/familial) were assessed using Chi-square and Fisher’s exact tests as appropriate. All analyses were conducted using SAS version 9.4 (SAS, Cary, NC).

## Results

Two hundred and forty patients met criteria for inclusion in the study. Of these, 153 (64%) had sPCT and 87 (36%) had fPCT (Table 1). The PCT population in this study is composed primarily of European Caucasians (94.6%) and consists of slightly more males (53.8%) than females (46.2%). (Table 1) The average age of diagnosis was 53.4 (±10.6). Excess ethanol consumption was significanlty associatated with cases of sPCT (83.7%) compared to fPCT (52.3%) (*p*<0.0001) as was HCV infection status [sPCT (63.3%) compared to fPCT (16.2%) (*p*<0.0001)]. (Table 1).

**Table 1:** Patient Demographics and Risk Factors for PCT.

	All PCT (n = 240)	Familial PCT (n = 87)	Sporadic PCT (n = 153)
	mean ± sd (no. observed)	mean ± sd (no. observed)	mean ± sd (no. observed)
Age at screening	53.4 ±10.6 (89)	50.4 ± 14.3 (25)	54.6 ± 8.6 (64)
	n/ no. observed (%)	n/ no. observed (%)	n/ no. observed (%)
Race/Ethnicity			
Caucasian	175/185 (94.6)	53/60 (88.3)	122/125 (97.6)
Native American	1/185 (0.5)	0	1/125 (0.8)
African American	2/185 (1.1)	1/60 (1.7)	1/125 (0.8)
Asian	2/185 (1.1)	2/60 (3.3)	0
Hispanic	5/185 (2.7)	4/60 (6.7)	1/125 (0.8)
Male	129/240 (53.8)	37/87 (42.5)	92/153 (60.1)
Excess Alcohol Consumption Prior to PCT Diagnosis^a^	117/161 (72.7)	30/57 (52.6)	87/104 (83.7)
HCV Positive	73/148 (49.3)	9/47 (19.1)	64/101 (63.4)

^a^ Ethanol consumption in excess of 20g per day

*HFE* sequence variants (C282Y and H63D) were significantly enriched among patients with both sPCT and fPCT (Table 2). Compared to the European (non-Finnish) EXAC population, GNPAT D519G was significantly enriched in patients with fPCT (*p* = 0.0014) but not in patients with sPCT (*p* = 0.4477) (Table 2) Within the two PCT populations, GNPAT D519G was significantly more frequent in fPCT (29.3%) compared to sPCT (18.0%) (*p* = 0.004). This enrichment in fPCT compared to sPCT was not observed for either HFE variant.

**Table 2:** Allele Frequency *of GNPAT D519G*, *HFE C282Y*, *and HFE H63D*.

Genoptype	HOM n (%)	HET n (%)	WT n (%)	Total n	Patient Frequency	Control Pop. Frequency^a^	*P*-value
**Familial PCT**							
GNPAT D519G	7 (8.1)	37 (42.5)	43 (49.4)	87	29.3	19.7	*p = 0*.*0014*
HFE H63D	2 (2.3)	33 (37.9)	52 (59.8)	87	21.3	13.7	*p = 0*.*0037*
HFE C282Y	3 (3.5)	21 (24.1)	63 (72.4)	87	15.5	5.1	*p<0*.*0001*
**Sporadic PCT**							
GNPAT D519G	0 (0)	55 (35.9)	98(64.1)	153	18.0	19.7	*p = 0*.*4477*
HFE H63D	11 (7.2)	48 (31.4)	94 (61.4)	153	22.9	13.7	*p<0*.*0001*
HFE C282Y	17 (11.1)	31 (20.3)	105 (68.6)	153	21.2	5.1	*p<0*.*0001*

^a^European (Non-Finnish) population frequency in Exome Aggregation Consortium database[19]

## Discussion

PCT is an iron dependent disease, and a genetic variant of HFE (C282Y) that enhances iron absorption and recycling by reducing hepcidin expression is a risk factor for PCT. The purpose of these studies was to investigate the hypothesis that GNPAT D519G is a risk factor for PCT. This hypothesis was based on observations by others that this polymorphism of GNPAT is enriched in male patients with type I hereditary hemochromatosis (*HFE* C282Y homozygotes) who present with a high iron phenotype, suggesting that like HFE, GNPAT is involved in regulation iron homeostasis. Our studies have shown that GNPAT D519G is enriched in patients with fPCT but not in sPCT. The basis of this difference is speculative but may be due to the relative potency of GNPAT D519G as an enhancer of iron absoption and recycling. Uroporphyrinogen III is the natural substrate of URO-D, however, when uroporphyrinogen III undergoes iron-dependent oxidation to uroporphomethene, it functions as a competitive inhibitor of the enzyme.[3] Given the relative abundance of hepatic iron, some formation of uroporphomethene likely occurs under normal physiological conditions, but the amount of inhibitor formed is insufficient to limit enzyme activity such that pathophysiologic levels of hepatic heme precursors accumulate. In cases of fPCT, however, where URO-D activity is abnormally low due to heterozygous mutation, even modest increases in hepatic iron concentration could result in inhibitor formation sufficient to produce clinical symptoms of PCT. Thus, a relatively weak modifier of iron metabolism, such as GNPAT D519G, would increase the probability of developing symptomatic PCT in individuals with fPCT (Table 2). On the other hand, relatively strong modifiers of iron metabolism such as HFE C282Y and H63D are more likely to generate the conditions necessary to produce symptomatic sPCT. (Table 2) The finding of a significantly greater frequency of excess alcohol consumption and HCV infection among patients with sPCT compared to those with fPCT (Table 1) supports the hypothesis that induction of the clinical phenotype in patients with sPCT requires a greater accumulation of disease-associated risk factors. In this case, excess alcohol consumption and HCV infection appears to enhance formation of the URO-D inhibitor by mediating oxidative liver injury.

The correlative relationship between *HFE* C282Y genotype and PCT in the current study supports our previous observations[7] and those of others[20] however, conflicting data surround the relationship between HFE H63D and PCT risk.[21] In a study involving fewer patients, we observed a non-significant trend toward enrichment of HFE H63D among patients with fPCT.[7] In that study, the frequency of HFE H63D was set at 15.9% (vs. 13.7% in the current study, Table 1). Some investigators have observed a higher frequency of HFE H63D among patients with PCT while others have not.[20, 22, 23] Those outcomes appear to be influenced by frequency estimates used for the control population. For example a study from France used a frequency of HFE H63D in the general population of 12.9% while a study from Brazil used a frequency of 31.1%.[22, 24] These disparities likely reflect differences in demographic characteristics of the study populations and sampling of relatively small numbers of individuals within the general population. The current study provides support for a correlative relationship between HFE H63D and PCT in the United States (Table 2).

The absence of HFE C282Y, HFE H63D or GNPAT D519G in 43 patients (17.8%), suggests that other genetic modifiers of iron metabolism may be identified in patients with PCT. Our studies demonstrate that GNPAT D519G is a risk factor for developing PCT when a *URO-D* mutation is also present. These results support both the characterization of PCT as an iron dependent disease and the concept of GNPAT D519G as a genetic modifier of iron homeostasis[25].

## Abbreviations

*GNPAT*: glyceronephosphate O-acyltransferase
*URO-D*: uroporphyrinogen decarboxylase
PCT: porphyria cutanea tarda
fPCT: familial PCT
sPCT: sporadic PCT
HCV: hepatitis C
